# The 2023 Impact of Inflammatory Bowel Disease in Canada: COVID-19 and IBD

**DOI:** 10.1093/jcag/gwad019

**Published:** 2023-09-05

**Authors:** Gilaad G Kaplan, M Ellen Kuenzig, Joseph W Windsor, Charles N Bernstein, Alain Bitton, Stephanie Coward, Jennifer L Jones, Kate Lee, Sanjay K Murthy, Laura E Targownik, Juan-Nicolás Peña-Sánchez, Sara Ghandeharian, Noelle Rohatinsky, Jake Weinstein, Tyrel Jones May, Mira Browne, Nazanin Jannati, Sahar Tabatabavakili, James H B Im, Saketh Meka, Sonya Vukovic, Tal Davis, Quinn Goddard, Julia Gorospe, Taylor Stocks, Léa Caplan, Najla Kanaan, Daniel Stuart, Tesa Ramsay, Kelly J Robinson, Diane Charron-Bishop, Eric I Benchimol

**Affiliations:** Departments of Medicine and Community Health Sciences, University of Calgary, Calgary, Alberta, Canada; SickKids Inflammatory Bowel Disease Centre, Division of Gastroenterology, Hepatology, and Nutrition, The Hospital for Sick Children, Toronto, Ontario, Canada; Child Health Evaluative Sciences, SickKids Research Institute, The Hospital for Sick Children, Toronto, Ontario, Canada; Departments of Medicine and Community Health Sciences, University of Calgary, Calgary, Alberta, Canada; Department of Internal Medicine, Max Rady College of Medicine, Rady Faculty of Health Sciences, University of Manitoba, Winnipeg, Manitoba, Canada; University of Manitoba IBD Clinical and Research Centre, Winnipeg, Manitoba, Canada; Division of Gastroenterology and Hepatology, McGill University Health Centre IBD Centre, McGill University, Montréal, Quebec, Canada; Departments of Medicine and Community Health Sciences, University of Calgary, Calgary, Alberta, Canada; Departments of Medicine, Clinical Health, and Epidemiology, Dalhousie University, Halifax, Nova Scotia, Canada; Crohn’s and Colitis Canada, Toronto, Ontario, Canada; Department of Medicine, University of Ottawa, Ottawa, Ontario, Canada; The Ottawa Hospital IBD Centre, Ottawa, Ontario, Canada; Division of Gastroenterology and Hepatology, Mount Sinai Hospital, University of Toronto, Toronto, Ontario, Canada; Department of Community Health and Epidemiology, University of Saskatchewan, Saskatoon, Saskatchewan, Canada; Crohn’s and Colitis Canada, Toronto, Ontario, Canada; College of Nursing, University of Saskatchewan, Saskatoon, Saskatchewan, Canada; SickKids Inflammatory Bowel Disease Centre, Division of Gastroenterology, Hepatology, and Nutrition, The Hospital for Sick Children, Toronto, Ontario, Canada; Child Health Evaluative Sciences, SickKids Research Institute, The Hospital for Sick Children, Toronto, Ontario, Canada; Division of Gastroenterology and Hepatology, University Health Network, University of Toronto, Toronto, Ontario, Canada; SickKids Inflammatory Bowel Disease Centre, Division of Gastroenterology, Hepatology, and Nutrition, The Hospital for Sick Children, Toronto, Ontario, Canada; Child Health Evaluative Sciences, SickKids Research Institute, The Hospital for Sick Children, Toronto, Ontario, Canada; Department of Community Health and Epidemiology, University of Saskatchewan, Saskatoon, Saskatchewan, Canada; Department of Gastroenterology, University of Toronto, Toronto, Ontario, Canada; SickKids Inflammatory Bowel Disease Centre, Division of Gastroenterology, Hepatology, and Nutrition, The Hospital for Sick Children, Toronto, Ontario, Canada; Child Health Evaluative Sciences, SickKids Research Institute, The Hospital for Sick Children, Toronto, Ontario, Canada; Department of Neuroscience, McGill University, Montreal, Quebec, Canada; Department of Internal Medicine, University of Toronto, Toronto, Ontario, Canada; SickKids Inflammatory Bowel Disease Centre, Division of Gastroenterology, Hepatology, and Nutrition, The Hospital for Sick Children, Toronto, Ontario, Canada; Child Health Evaluative Sciences, SickKids Research Institute, The Hospital for Sick Children, Toronto, Ontario, Canada; Departments of Medicine and Community Health Sciences, University of Calgary, Calgary, Alberta, Canada; Departments of Medicine and Community Health Sciences, University of Calgary, Calgary, Alberta, Canada; Crohn’s and Colitis Canada, Toronto, Ontario, Canada; Departments of Medicine and Community Health Sciences, University of Calgary, Calgary, Alberta, Canada; Crohn’s and Colitis Canada, Toronto, Ontario, Canada; Crohn’s and Colitis Canada, Toronto, Ontario, Canada; Crohn’s and Colitis Canada, Toronto, Ontario, Canada; Crohn’s and Colitis Canada, Toronto, Ontario, Canada; Crohn’s and Colitis Canada, Toronto, Ontario, Canada; SickKids Inflammatory Bowel Disease Centre, Division of Gastroenterology, Hepatology, and Nutrition, The Hospital for Sick Children, Toronto, Ontario, Canada; Child Health Evaluative Sciences, SickKids Research Institute, The Hospital for Sick Children, Toronto, Ontario, Canada; Department of Paediatrics, Temerty Faculty of Medicine, University of Toronto, Toronto, Ontario, Canada; ICES, Toronto, Ontario, Canada; Institute of Health Policy, Management, and Evaluation, Dalla Lana School of Public Health, University of Toronto, Toronto, Ontario, Canada

**Keywords:** Crohn’s disease, -Ulcerative colitis, SARS-CoV-2, Coronavirus

## Abstract

The COVID-19 pandemic had a monumental impact on the inflammatory bowel disease (IBD) community. At the beginning of the pandemic, knowledge on the effect of SARS-CoV-2 on IBD was lacking, especially in those with medication-suppressed immune systems. Throughout the pandemic, scientific literature exponentially expanded, resulting in clinical guidance and vaccine recommendations for individuals with IBD. Crohn’s and Colitis Canada established the COVID-19 and IBD Taskforce to process and communicate rapidly transforming knowledge into guidance for individuals with IBD and their caregivers, healthcare providers, and policy makers. Recommendations at the onset of the pandemic were based on conjecture from experience of prior viruses, with a precautionary principle in mind. We now know that the risk of acquiring COVID-19 in those with IBD is the same as the general population. As with healthy populations, advanced age and comorbidities increase the risk for severe COVID-19. Individuals with IBD who are actively flaring and/or who require high doses of prednisone are susceptible to severe COVID-19 outcomes. Consequently, sustaining maintenance therapies (e.g., biologics) is recommended. A three-dose mRNA COVID-19 vaccine regimen in those with IBD produces a robust antibody response with a similar adverse event profile as the general population. Breakthrough infections following vaccine have been observed, particularly as the virus continues to evolve, which supports receiving a bivalent vaccine booster. Limited data exist on the impact of IBD and its therapies on long-term outcomes following COVID-19. Ongoing research is necessary to address new concerns manifesting in those with IBD throughout the evolving pandemic.

## Teaser

In April 2021, Crohn’s and Colitis Canada’s COVID-19 and IBD Taskforce produced the 2021 Impact of COVID-19 & Inflammatory Bowel Disease in Canada. This report summarized the key learnings of the impact of COVID-19 on the IBD community at the one-year anniversary since the COVID-19 pandemic was declared by the World Health Organization. During the first year of the pandemic, and before the widespread dissemination of COVID-19 vaccines, the body of the scientific literature was synthesized and communicated to the IBD community through Crohn’s and Colitis Canada’s COVID-19 and IBD Taskforce using online resources and a webinar series. The scientific literature from that time period suggested that those with IBD shared a similar risk of becoming infected by SARS-CoV-2 or experiencing severe outcomes as the general population with the primary exception being those flaring and/or requiring high doses of oral systemic corticosteroids. During the first year, lockdowns and shielding populations believed to be at high risk, like the IBD community, reduced the risk of transmission. However, mental health distress was prevalent due to isolation and uncertainty of risks, which was exacerbated in immunocompromised individuals with IBD. Major breakthroughs in novel SARS-CoV-2 vaccines and antiviral therapeutics, as well as adaptation to models of healthcare delivery (e.g., virtual care) offered hope that the second year of the pandemic would realize the easing of restrictions.

**Key Points**

1. Crohn’s and Colitis Canada’s COVID-19 and IBD Taskforce compiled knowledge and communicated with the IBD community through an expert-generated website and frequent webinars for a public audience. This work has set the stage for Crohn’s and Colitis Canada to inform the IBD community in the event of any future public health emergencies.
2. Infection of SARS-CoV-2 for those with IBD is similar to the general population. Those with IBD are not at increased risk of acquiring COVID-19 or experiencing severe COVID-19 outcomes.
3. Persons with IBD who require a high dose (>20 mg/day) of corticosteroids are at higher risk of experiencing severe complications from COVID-19.
4. Those with IBD under the age of 50 who are actively flaring are at higher risk of severe COVID-19 outcomes.
5. Maintenance therapies (e.g., biologics) for IBD are not associated with more serious disease course following infection from SARS-CoV-2.
6. Vaccines for COVID-19 are safe and elicit robust immune responses to SARS-CoV-2 infection following three doses of vaccine in individuals with IBD, although immunity is less robust among those receiving prednisone to treat a flare or those on anti-TNF treatments.
7. As breakthrough infections following vaccination occur at an increasing rate for subvariants of Omicron, a booster dose with a bivalent vaccine is recommended for those with IBD.
8. Guidance to the IBD community consistently recommended to continue IBD therapies during the pandemic to avoid flaring from lack of adherence.

## INTRODUCTION: CROHN’S AND COLITIS CANADA’S COVID-19 & IBD TASKFORCE

Inflammatory bowel disease (IBD) affects more than 0.8% of the Canadian population in 2023 (1–4). When the World Health Organization declared COVID-19 a global pandemic on March 11, 2020, immunocompromised individuals with IBD were initially considered vulnerable to infection and complication by SARS-CoV-2 (5). As the SARS-CoV-2 virus was novel, direct clinical evidence was lacking to inform healthcare providers and policy makers on guidance for immunocompromised individuals. Consequently, Crohn’s and Colitis Canada developed the COVID-19 and IBD Taskforce on March 12, 2020 (6). The Taskforce included adult and paediatric gastroenterologists from across the country with infectious disease specialists, nurses, and patient representatives. This team met regularly to review the emerging evidence on the impact of COVID-19 on those with IBD to establish recommendations for the IBD community (6).

Expert reviews of population-level recommendations were tailored to the IBD community and communicated through website FAQs and infographics (6); a public-oriented burden report (7) with foci on additional special populations (e.g., pregnant people (8), pediatrics (8), seniors (9)), IBD medications (10), mental health (11), and access to care (12); and through a moderated, online webinar series. The one- to two-hour webinar recordings were then curated into three- to five-minute video clips to answer specific questions and uploaded to Crohn’s and Colitis Canada’s YouTube page (6). As of October 2022, Crohn’s and Colitis Canada’s webpage on COVID-19 has been viewed over 800,000 times, and the thirty webinars produced on COVID-19 and IBD were viewed over 81,000 times.

Crohn’s and Colitis Canada’s 2021 Impact of COVID-19 and IBD report synthesized the knowledge learned by the COVID-19 and IBD Taskforce (13). The purpose of this article is to provide up-to-date knowledge on the influence of COVID-19 on the IBD community.

### Epidemiology: The Risk of COVID-19 among those with IBD is Similar to the General Population

The global pandemic was in part driven by the high transmission of SARS-CoV-2. Earlier in the pandemic, concern was raised that those with IBD might be at higher risk of being infected by SARS-CoV-2. The Surveillance Epidemiology of Coronavirus Under Research Exclusion (SECURE-IBD) registry is an international cohort study that recruited over 6000 individuals with IBD who were diagnosed with COVID-19 (14). Analyzing the waves of reporting into the SECURE-IBD registry showed similar patterns to the general population during the first year of the pandemic, which provided the first clues that having IBD or being immunosuppressed by therapies to treat IBD may not increase the risk of acquiring SARS-CoV-2 (15). Subsequently, a meta-analysis of seven observational studies showed that individuals with IBD had comparable rates of COVID-19 as the general population (pooled odds ratio [OR]: 0.47; 95% CI: 0.18, 1.26) (16). However, additional studies are necessary to assess whether the risk of SARS-CoV-2 was influenced by public health recommendations geared towards immunocompromised populations and adherence among those with IBD (6).

### General Risk: Those with IBD had Similar Risk Factors for Severe COVID-19 with the General Population

Overall, the accumulating data consistently demonstrated that the risk of severe COVID-19 in the general population was similar to those with Crohn’s disease or ulcerative colitis (16). Moreover, the risk factors associated with severe COVID-19, defined as hospitalization or death, were similar in those with IBD as compared to the general population: namely age and comorbidities (16–19). Like the general population, seniors with IBD (particularly those with multiple comorbidities such as diabetes, cancer, and cardiovascular disease) were at the highest risk for hospitalization or death from SARS-CoV-2 infection (16–18).

### IBD Risk—Individuals on Prednisone were at Risk for Severe COVID-19 Outcomes

Numerous studies assessed the risk of severe COVID-19 in relation to the medications used to treat IBD. By July 2020, the first 500 cases reported in SECURE-IBD provided clues on the risk of drugs on severe COVID-19 (20). In 2022, the largest study—including 6000 individuals with IBD from March 2020 to May 2021 (before widespread access to vaccines)—reported that those with IBD who were using anti-TNFs, vedolizumab, ustekinumab, or tofacitinib at the time of their infection with SARS-CoV-2 had a lower risk of hospitalization or death from COVID-19 (21). In contrast, those who experienced an active flare and required higher doses of oral prednisone (>20 mg/day), were more likely to experience severe outcomes of from COVID-19 (20–22). The risk of severe COVID-19 was particularly observed among individuals under the age of 50 who were flaring with IBD (22). Consequently, guidance to the IBD community consistently recommended to continue IBD therapies during the pandemic to avoid flaring from lack of adherence. Moreover, those with IBD who were flaring were recommended to isolate while on high doses of prednisone (10). Unlike medical therapy, the risk of severe COVID-19 is not specifically increased among those who had prior surgery for IBD (23). Among those with IBD who are at increased risk for severe COVID-19, treatment with Paxlovid (nirmatrelvir and ritonavir) reduced the risk of hospitalization as compared to those not treated with antiviral therapy (24).

### Individuals with IBD Require Regular Booster Doses of SARS-CoV-2 Vaccines to Maintain Immunity

The approval of the first mRNA and nonreplicating viral vector vaccines against SARS-CoV-2 occurred in December 2020 (25). Throughout 2021, numerous studies were conducted to evaluate the serological response to different dose regimens of the COVID-19 vaccines among immunocompromised individuals with IBD. The largest serological study in people with IBD is CLARITY-IBD, which initially showed that the antibody response following a two-dose vaccine schedule is superior for mRNA vaccines compared to adenovirus vector vaccines, for those on vedolizumab compared to infliximab, and those on infliximab monotherapy versus those on concomitant immunomodulator therapy (26). The VIP study demonstrated a lower antibody response after two vaccine doses among those using infliximab and tofacitinib, those who receive a vector adenovirus vaccine compared to an mRNA, and those of advanced age (27). A meta-analysis of 46 studies in the IBD population confirmed high seroconversion (96%) after completing a two-dose vaccine series, with lower serological response in those on anti-TNF therapies, and a subsequent decay of antibodies over time (28). Adolescents with IBD mount a robust antibody response to a two-dose mRNA vaccine regimen (29,30).

**Figure 1:**
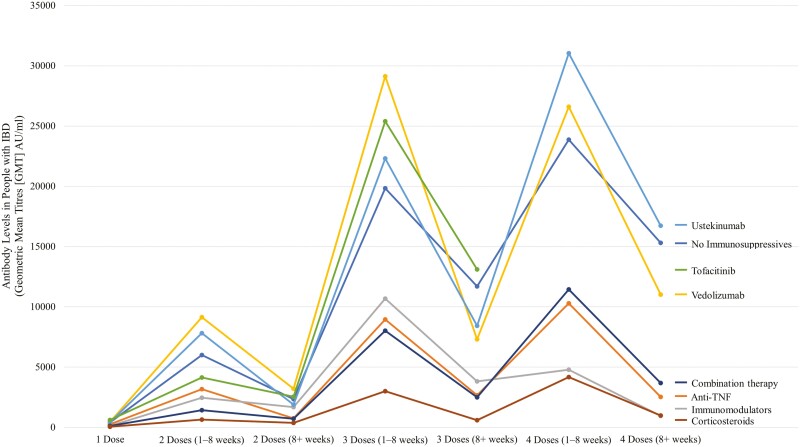
Average antibodies by time period relative to vaccine doses in a cohort of 556 individuals with IBD from Calgary, Alberta, Canada, grouped by IBD medication class. Adapted from Quan *et al.* 2023 (39).

A large prospective cohort study in Calgary, Alberta established the immunological efficacy of a three-dose mRNA vaccine series for those with IBD (31). Following the third vaccine dose, the seroconversion rate was 99.6% with a high geometric mean titer within eight weeks of the third dose; however, after eight weeks, antibody levels fell by approximately 12% (95% CI: 8%, 15%) per week (31). The independent factors associated with a reduced serological response were advanced age and use of oral prednisone at the time of the third vaccine dose (Figure 1) (31). Similarly, the CLARITY-IBD and VIP studies demonstrated robust serological responses following a third SARS-CoV-2 vaccine, though anti-TNF therapy and tofacitinib were associated with lower antibody levels (32,33). These data have led various jurisdictions to recommend a fourth vaccine dose, which has been associated with recapturing decaying antibody levels in those with IBD (34). Despite booster doses of SARS-CoV-2 mRNA vaccines advocated by Crohn’s and Colitis Canada (35), data from Ontario indicated low uptake of a third vaccine dose in the IBD population in the pre-Omicron era (i.e., before December 2021) (36).

### Bivalent Vaccines are Recommended for those with IBD due to Breakthrough Infections

Prior to the Omicron variant becoming the dominant strain, before December 2021, breakthrough infections following the completion of a vaccine series in those with IBD were not common. In a large cohort study in the United States, completed before December 2021, only 1.7% of individuals with IBD reported COVID-19 more than one month after completing their vaccine series (37). Moreover, individuals with a breakthrough infection had lower mean antibody levels (37). Similarly, a meta-analysis conducted on studies prior to the Omicron era showed the pooled risk of breakthrough infections after two vaccine doses was ~1%, and the risk of breakthrough infections in those with IBD was similar to the general population (pooled relative risk [RR]: 0.60; 95% CI: 0.25, 1.42) (38).

Studies conducted during the Omicron wave (December 2021–present); however, have reported considerably higher breakthrough infection rates in those with IBD due to the highly infectious transmissibility and vaccine evasive subvariants of Omicron. The CLARITY-IBD study demonstrated that, during the Omicron wave, 15% of individuals with IBD experienced a breakthrough infection after a three-dose SARS-CoV-2 vaccine regimen (32). Although breakthrough infections occurred more commonly in those on infliximab compared to vedolizumab, antibody levels alone were not associated with protection from infection (32). These data suggest that those with IBD will likely benefit from bivalent vaccines (i.e., mRNA of native spike protein with subvariant: BA.1, BA.4, and/or BA.5). Moreover, a robust serological response to the bivalent vaccines has been shown in the IBD population (39), though the durability over time will need to be confirmed in future studies.

### SARS-CoV-2 Vaccines are Safe in Those with IBD and do not Trigger a Flare of Disease Activity

Overall, the short-term adverse events to SARS-CoV-2 vaccines in those with IBD are similar to the general population. A cohort study from the US showed that the most commonly reported adverse events after receiving a vaccine were: injection site pain, fatigue, and malaise; the rate of reporting in those with IBD was not different than healthy controls (40). Adverse events were classified as mild and short-lived (i.e., less than two days). After the third dose of a vaccine, approximately 41% of people with IBD reported an adverse event. Most adverse events decreased in severity from second to third dose (40). The one exception was gastrointestinal symptoms, which were slightly worse after the third dose of the vaccine (40). However, a Canadian study on adverse events within 30 days of each vaccine dose documented no objective risk of flaring IBD within 30 days of receiving either the first, second, or third vaccine dose (41).

### Complications of COVID-19 in Those with IBD Include Mental Health Concerns and Possible Long COVID

Overall, the long-term complications of COVID-19 in those with IBD appear to be similar to the general population (42); though, high quality, longitudinal studies in the IBD population are lacking. Post acute COVID-19 syndrome (also known as long COVID-19) is a consideration for those with IBD as SARS-CoV-2 antigens may persists in intestinal mucosa for months after clearing the infection (43). Statistics Canada reported 14.8% of Canadians experience symptoms of long COVID three months after infection; population-based data from Ontario indicated increased health services utilization following COVID-19; and individuals with long COVID demonstrate physiological changes (44,45). However, studies in the IBD population are necessary to assess if the risk is elevated of post acute COVID-19 syndrome (46). Irrespective of whether individuals with IBD are at additional risk of long COVID compared to the general population, overlapping long COVID and IBD places a significant burden on people living with IBD and the health systems.

Deterioration in mental health and elevated stress and anxiety impacted the IBD community as many feared worse outcomes from COVID-19 (11,47). Depressive symptoms and distress were exacerbated in those who experienced isolation in an attempt to shield themselves from exposure to SARS-CoV-2 (11,47). Furthermore, stress and anxiety were associated with worsening of gastrointestinal symptoms and disease activity (see also Graff *et al.* this volume) (48). Consequently, many individuals with IBD struggled with low health-related quality of life during the pandemic (49), which was also observed in children with IBD (50).

## Conclusion

Crohn’s and Colitis Canada’s COVID-19 and IBD Taskforce synthesized the medical literature during the pandemic in order to communicate timely and insightful guidance to the IBD community. Over the last three years, since the onset of the pandemic, the knowledge on the impact of COVID-19 on those with IBD has expanded dramatically. The risk of COVID-19 among those with IBD is similar to the general population. Individuals with IBD had similar risk factors for severe COVID-19 as the general population, namely age and comorbidities. Those with IBD who flared and were administered prednisone were at risk for severe COVID-19 outcomes. Vaccines served as the primary preventative health measure. Individuals with IBD require at least a three dose SARS-CoV-2 vaccine regimen. In addition, bivalent vaccines are recommended due to breakthrough infections and immune-escaping variants of the virus. Importantly, SARS-CoV-2 vaccines are safe in those with IBD and do not trigger a flare of disease activity. Data on the long-term impact of COVID-19 in people with IBD are lacking. Despite actions to guide the IBD community through the pandemic, those with IBD struggled with mental health concerns and impaired quality of life during the pandemic.

## Knowledge Gap & Future Research Directions

1. There is still work to be done to understand the impact of public health recommendations geared to immunocompromised populations on the risk of infection by SARS-CoV-2.
2. Determining the vaccine regimen that provides robust and durable serological response in those with IBD who are immunocompromised requires ongoing research.
3. Exploring the effect of bivalent vaccines in reducing breakthrough infections following vaccination will be important for future studies.
4. Determining the mental health sequelae and risk of long-term sequelae of COVID-19 (Long COVID Syndrome) in those with IBD is necessary.
5. Lessons learned from the COVID-19 pandemic to prepare the IBD community for future viral outbreaks should be documented and incorporated into emergency planning measures.

## Patient & Caregiver Partner Perspective

Patient partners emphasized that the risks of acquiring COVID-19 and the outcomes of COVID-19 are similar in individuals with IBD as they are for the general population. Preventing disease exacerbations and the need for high-dose steroid use are important to avoid more severe complications related to COVID-19. Partners stressed the importance of continuing with IBD-related medication therapies to prevent disease flare-ups and the need for steroid use. COVID-19 vaccines were recognized as safe for individuals with IBD. Encouragement should be provided to individuals with IBD to receive bivalent vaccine boosters to allow for continued protection against COVID-19. Information provided in this article provides peace of mind and reassurance to patient partners, allowing them to make educated decisions about their physical and mental wellbeing. Patient partners encouraged greater access to mental health supports for pandemic-related isolation, stress, and anxiety that many individuals experienced. Partners recognized there were some individuals, such as those in active flares and/or on >20 mg/day of prednisone, who remain clinically vulnerable or who may experience increased vulnerability to worse COVID-19 outcomes if they contract the virus. Ongoing protection and advocacy efforts need to center around these clinically vulnerable individuals. It was recommended that individuals perform their own risk assessment based on the scientific information available.

## Policy Implications & Key Advocacy Outcomes

1. Crohn’s and Colitis Canada’s COVID-19 and IBD Taskforce served as an invaluable resource to synthesize rapidly evolving information on the pandemic and communicate this knowledge to the IBD community via online sites and webinar series.
2. Crohn’s and Colitis Canada can educate healthcare providers on the appropriate guidance of managing IBD throughout the pandemic, especially on the importance of receiving bivalent vaccines.
3. Crohn’s and Colitis Canada should continue to advocate to policymakers and health authorities during the pandemic for vulnerable immunocompromised individuals with IBD.

## Data Availability

No new data were generated or analyzed in support of this review.

## References

[1] Coward S , Clement F, Benchimol EI, et al. Past and future burden of inflammatory bowel diseases based on modeling of population-based data. Gastroenterology. 2019;156:1345–1353.e4. doi:10.1053/j.gastro.2019.01.002. 30639677

[2] Kaplan GG , Windsor JW. The four epidemiological stages in the global evolution of inflammatory bowel disease. Nat Rev Gastroenterol Hepatol. 2021;18:56–66. doi:10.1038/s41575-020-00360-x. 33033392 PMC7542092

[3] Molodecky NA , Soon IS, Rabi DM, et al. Increasing incidence and prevalence of the inflammatory bowel diseases with time, based on systematic review. Gastroenterology. 2012;142:46–54.e42; quiz e30. doi:10.1053/j.gastro.2011.10.001. quize30. 22001864

[4] Ng SC , Shi HY, Hamidi N, et al. Worldwide incidence and ­prevalence of inflammatory bowel disease in the 21st century: a systematic review of population-based studies. Lancet. 2017;390:2769–78. doi:10.1016/S0140-6736(17)32448-0. 29050646

[5] Rubin DT , Abreu MT, Rai V, Siegel CA; International Organization for the Study of Inflammatory Bowel Disease. Management of patients with Crohn’s disease and ulcerative Colitis during the coronavirus disease-2019 pandemic: results of an International Meeting. Gastroenterology. 2020;159:6–13.e6. doi:10.1053/j.gastro.2020.04.002. 32272113 PMC7194599

[6] Kaplan GG , Windsor JW, Crain J, et al. Crohn’s and Colitis Canada’s 2021 impact of COVID-19 & inflammatory bowel disease in Canada: a knowledge translation strategy. J Can Assoc Gastroenterol. 2021;4:S10–9. doi:10.1093/jcag/gwab028. 34755034 PMC8570425

[7] Ellen Kuenzig M , Windsor JW, Barrett L, et al. Crohn’s and Colitis Canada’s 2021 impact of COVID-19 and inflammatory bowel disease in Canada: executive summary. J Can Assoc Gastroenterol. 2021;4:S1–9. doi:10.1093/jcag/gwab027. 34755033 PMC8570424

[8] Benchimol EI , Carroll MW, Geist R, et al. Crohn’s and Colitis Canada’s 2021 impact of COVID-19 and inflammatory bowel disease in Canada: children and expectant mothers with inflammatory bowel disease. J Can Assoc Gastroenterol. 2021;4:S27–33. doi:10.1093/jcag/gwab030. 34755036 PMC8570420

[9] Bernstein CN , Singh H, Murthy SK, et al. Crohn’s and Colitis Canada’s 2021 impact of COVID-19 and inflammatory bowel disease in Canada: seniors with IBD. J Can Assoc Gastroenterol. 2021;4:S34–9. doi:10.1093/jcag/gwab025. 34755037 PMC8570427

[10] Targownik LE , Bernstein CN, Lakatos PL, et al. Crohn’s and Colitis Canada’s 2021 impact of COVID-19 and inflammatory bowel disease in Canada: risk factors and medications. J Can Assoc Gastroenterol. 2021;4:S40–5. doi:10.1093/jcag/gwab032. 34755038 PMC8570417

[11] Graff LA , Fowler S, Jones JL, et al. Crohn’s and Colitis Canada’s 2021 impact of COVID-19 and inflammatory bowel disease in Canada: mental health and quality of life. J Can Assoc Gastroenterol. 2021;4:S46–53. doi:10.1093/jcag/gwab031. 34755039 PMC8570421

[12] Jones JL , Benchimol EI, Bernstein CN, et al. Crohn’s and Colitis Canada’s 2021 impact of COVID-19 and inflammatory bowel disease in Canada: health care delivery during the pandemic and the future model of inflammatory bowel disease care. J Can Assoc Gastroenterol. 2021;4:S61–7. doi:10.1093/jcag/gwab034. 34755041 PMC8570426

[13] Kuenzig EM , Windsor JW, Barrett L, et al. Crohn’s and Colitis Canada’s 2021 impact of COVID-19 and inflammatory bowel disease in Canada: executive summary. J Can Assoc Gastroenterol. 2021;4:S1–9. 34755033 10.1093/jcag/gwab027 PMC8570424

[14] Windsor JW , Underwood FE, Brenner E, et al. Data visualization in the era of COVID-19: an interactive map of the SECURE-IBD registry. Am J Gastroenterol. 2020;115:1923–4. doi:10.14309/ajg.0000000000000953. 33156119

[15] Kaplan GG , Underwood FE, Coward S, et al. The multiple waves of covid-19 in patients with inflammatory bowel disease: a temporal trend analysis. Inflamm Bowel Dis. 2022;28(11):1687–95. 35032167 10.1093/ibd/izab339 PMC8807298

[16] Singh AK , Jena A, Kumar MP, et al. Risk and outcomes of coronavirus disease in patients with inflammatory bowel disease: A systematic review and meta-analysis. United Eur Gastroenterol J. 2021;9:159–76. 10.1177/2050640620972602 PMC8250629 33210980

[17] Attauabi M , Poulsen A, Theede K, et al. Prevalence and outcomes of COVID-19 among patients with inflammatory bowel disease: a Danish prospective population-based cohort study. J Crohns Colitis. 2021;15:540–50. doi:10.1093/ecco-jcc/jjaa205. 33035299 PMC7797764

[18] Ludvigsson JF , Axelrad J, Halfvarson J, et al. Inflammatory bowel disease and risk of severe COVID-19: A nationwide population-based cohort study in Sweden. United Eur Gastroenterol J. 2021;9:177–92. doi:10.1002/ueg2.12049. PMC8014882 33704918

[19] Parekh R , Zhang X, Ungaro RC, et al. Presence of comorbidities associated with severe coronavirus infection in patients with inflammatory bowel disease. Dig Dis Sci. 2022;67:1271–7. doi:10.1007/s10620-021-07104-0. 34181165 PMC8237780

[20] Brenner EJ , Ungaro RC, Gearry RB, et al. Corticosteroids, but not TNF antagonists, are associated with adverse COVID-19 outcomes in patients with inflammatory bowel diseases: results from an international registry. Gastroenterology. 2020;159:481–491.e3. doi:10.1053/j.gastro.2020.05.032. 32425234 PMC7233252

[21] Ungaro RC , Brenner EJ, Agrawal M, Zhang X, Kappelman MD, Colombel J-F; Surveillance Epidemiology of Coronavirus Under Research Exclusion for Inflammatory Bowel Disease (SECURE-IBD) Research Group. Impact of medications on COVID-19 outcomes in inflammatory bowel disease: analysis of more than 6000 patients from an international registry. Gastroenterology. 2022;162:316–319.e5. doi:10.1053/j.gastro.2021.09.011. 34529987 PMC8437703

[22] Ricciuto A , Lamb CA, Benchimol EI, et al. Inflammatory bowel disease clinical activity is associated with COVID-19 severity especially in younger patients. J Crohns Colitis. 2022;16:591–600. doi:10.1093/ecco-jcc/jjab172. 34570886 PMC8522422

[23] Remzi FH , Panis Y, Spinelli A, et al. International organization for the study of IBD recommendations for surgery in patients with IBD during the coronavirus disease 2019 pandemic. Dis Colon Rectum. 2020;63:870–3. doi:10.1097/DCR.0000000000001718. 32355056

[24] Hashash JG , Desai A, Kochhar GS, et al. Efficacy of paxlovid and lagevrio for COVID-19 infection in patients with inflammatory bowel disease: a propensity-matched study. Clin Gastroenterol Hepatol. 2022;21(3):841–3. 36152898 10.1016/j.cgh.2022.09.011 PMC9492393

[25] Krammer F. SARS-CoV-2 vaccines in development. Nature. 2020;586:516–27. doi:10.1038/s41586-020-2798-3. 32967006

[26] Kennedy NA , Lin S, Goodhand JR, et al.; Contributors to the CLARITY IBD study. Infliximab is associated with attenuated immunogenicity to BNT162b2 and ChAdOx1 nCoV-19 SARS-CoV-2 vaccines in patients with IBD. Gut. 2021;70:1884–93. doi:10.1136/gutjnl-2021-324789. 33903149

[27] Alexander JL , Kennedy NA, Ibraheim H, et al.; VIP study investigators. COVID-19 vaccine-induced antibody responses in immunosuppressed patients with inflammatory bowel disease (VIP): a multicentre, prospective, case-control study. Lancet Gastroenterol Hepatol. 2022;7:342–52. doi:10.1016/S2468-1253(22)00005-X. 35123676 PMC8813209

[28] Jena A , James D, Singh AK, Dutta U, Sebastian S, Sharma V. Effectiveness and durability of COVID-19 vaccination in 9447 patients with ibd: a systematic review and meta-analysis. Clin Gastroenterol Hepatol. 2022;20:1456–1479.e18. doi:10.1016/j.cgh.2022.02.030. 35189387 PMC8856753

[29] Bronsky J , Copova I, Durilova M, et al. Post-vaccination immunogenicity of BNT162b2 SARS-CoV-2 vaccine and its predictors in pediatric inflammatory bowel disease. J Pediatr Gastroenterol Nutr. 2023;76(2):e36–44 doi:10.1097/MPG.0000000000003661. PMC9847686 36705698

[30] Shire ZJ , Reicherz F, Lawrence S, et al. Antibody response to the BNT162b2 SARS-CoV-2 vaccine in paediatric patients with inflammatory bowel disease treated with anti-TNF therapy. Gut. 2022;71:1922–4. doi:10.1136/gutjnl-2021-326196. 34815272

[31] Quan J , Ma C, Panaccione R, et al. Serological responses to three doses of SARS-CoV-2 vaccination in inflammatory bowel disease. Gut, 2023;72:802–4. 35606090 10.1136/gutjnl-2022-327440 PMC10086278

[32] Kennedy NA , Janjua M, Chanchlani N, et al. Vaccine escape, increased breakthrough and reinfection in infliximab-treated patients with IBD during the Omicron wave of the SARS-CoV-2 pandemic. Gut. 2023;72:295–305. 35902214 10.1136/gutjnl-2022-327570

[33] Alexander JL , Liu Z, Muñoz Sandoval D, et al.; VIP study investigators. COVID-19 vaccine-induced antibody and T-cell responses in immunosuppressed patients with inflammatory bowel disease after the third vaccine dose (VIP): a multicentre, prospective, case-control study. Lancet Gastroenterol Hepatol. 2022;7:1005–15. doi:10.1016/S2468-1253(22)00274-6. 36088954 PMC9458592

[34] Quan J , Ma C, Panaccione R, et al.; STOP COVID-19 in IBD Research Group. Serological responses to the first four doses of SARS-CoV-2 vaccine in patients with inflammatory bowel disease. Lancet Gastroenterol Hepatol. 2022;7:1077–9. doi:10.1016/S2468-1253(22)00340-5. 36306801 PMC9597523

[35] Murthy SK , Kuenzig ME, Windsor JW, et al. Crohn’s and Colitis Canada’s 2021 impact of COVID-19 and inflammatory bowel disease in Canada: COVID-19 vaccines-biology, current evidence and recommendations. J Can Assoc Gastroenterol. 2021;4:S54–60. doi:10.1093/jcag/gwab033. 34755040 PMC8570416

[36] Kuenzig ME , Widdifield J, Bernatsky S, Kaplan GG, Benchimol EI. Uptake of third doses of SARS-CoV-2 vaccines among people with inflammatory bowel disease in Ontario, Canada. Lancet Gastroenterol Hepatol. 2022;7:288–9. doi:10.1016/S2468-1253(22)00054-1. PMC8863433 35216658

[37] Weaver KN , Zhang X, Dai X, et al. Low rates of breakthrough COVID-19 infection after SARS-CoV-2 vaccination in patients with inflammatory bowel disease. Inflamm Bowel Dis. 2023;29(3):483–6. 35830416 10.1093/ibd/izac138 PMC9384490

[38] Jena A , James D, Singh AK, et al. Effectiveness and durability of COVID-19 vaccination in 9447 patients with ibd: a systematic review and meta-analysis. Clin Gastroenterol Hepatol. 2022;20(7):1456–79. 35189387 10.1016/j.cgh.2022.02.030 PMC8856753

[39] Quan J , Markovinovic A, Ma C, et al. Bivalent mRNA SARS-CoV-2 vaccination yields a strong serological response that is comparable to fourth dose in patients with inflammatory bowel disease. Gastroenterology. In Press.

[40] Li D , Debbas P, Mujukian A, et al. Postvaccination symptoms after a third dose of mRNA SARS-CoV-2 vaccination in patients with inflammatory bowel disease: results from CORALE-IBD. Inflamm Bowel Dis. 2022. 10.1093/ibd/izac174 PMC9452161 35998072

[41] Markovinovic A , Herauf M, Quan J, et al. Adverse events and serological responses following SARS-CoV-2 vaccination in individuals with inflammatory bowel disease. Am J Gastroenterol. 2022;117:e727–8. doi:10.14309/01.ajg.0000860656.63502.70. PMC10453345 37216598

[42] Zabana Y , Marín-Jiménez I, Rodríguez-Lago I, et al. Nationwide COVID-19-EII study: incidence, environmental risk factors and long-term follow-up of patients with inflammatory bowel disease and COVID-19 of the ENEIDA registry. J Clin Med. 2022;11:421. doi:10.3390/jcm11020421. 35054116 PMC8781643

[43] Zollner A , Koch R, Jukic A, et al. Postacute COVID-19 is characterized by gut viral antigen persistence in inflammatory bowel diseases. Gastroenterology. 2022;163:495–506.e8. doi:10.1053/j.gastro.2022.04.037. 35508284 PMC9057012

[44] McNaughton CD , Austin PC, Sivaswamy A, et al. Post-acute health care burden after SARS-CoV-2 infection: a retrospective cohort study. CMAJ. 2022;194:E1368–76. doi:10.1503/cmaj.220728. 36252983 PMC9616149

[45] Canada S. Long-term symptoms in Canadian adults who tested positive for COVID-19 or suspected an infection, January 2020 to August 2022. Volume 2022, 2022: https://www150.statcan.gc.ca/n1/daily-quotidien/221017/dq221017b-eng.htm.

[46] Meringer H , Mehandru S. Gastrointestinal post-acute COVID-19 syndrome. Nat Rev Gastroenterol Hepatol. 2022;19:345–6. doi:10.1038/s41575-022-00611-z. 35383321 PMC8981882

[47] D’Amico F , Rahier JF, Leone S, Peyrin-Biroulet L, Danese S. Views of patients with inflammatory bowel disease on the COVID-19 pandemic: a global survey. Lancet Gastroenterol Hepatol. 2020;5:631–2. doi:10.1016/S2468-1253(20)30151-5. 32411920 PMC7220169

[48] Goodday SM , Travis S, Walsh A, Friend SH. Stress-related consequences of the coronavirus disease 2019 pandemic on symptoms of Crohn’s disease. Eur J Gastroenterol Hepatol. 2021;33:1511–6. doi:10.1097/MEG.0000000000002081. 33512845 PMC8555884

[49] de Bock E , Filipe MD, Meij V, et al. Quality of life in patients with IBD during the COVID-19 pandemic in the Netherlands. BMJ Open Gastroenterol. 2021;8:e000670. doi:10.1136/bmjgast-2021-000670. PMC8257293 34215570

[50] Fedele F , Martinelli M, Strisciuglio C, et al. Health related quality of life in pediatric Inflammatory bowel disease during COVID-19 pandemic: a prospective study. J Pediatr Gastroenterol Nutr. 2022;75(5):595–600. 35897141 10.1097/MPG.0000000000003576

